# Silymarin as a Natural Antioxidant: An Overview of the Current Evidence and Perspectives

**DOI:** 10.3390/antiox4010204

**Published:** 2015-03-20

**Authors:** Peter F. Surai

**Affiliations:** 1 Department of Microbiology and Biochemistry, Faculty of Veterinary Medicine, Trakia University, Stara Zagora 6000, Bulgaria; E-Mail: psurai@feedfood.co.uk; Tel.: +44-7545-556-336; Fax: +44-1292-880-412; 2 Department of Animal Nutrition, Faculty of Agricultural and Environmental Sciences, Szent Istvan University, Gödöllo H-2103, Hungary; 3 Department of Veterinary Expertise and Microbiology, Faculty of Veterinary Medicine, Sumy National Agrarian University, Sumy 40021, Ukraine; 4 Odessa National Academy of Food Technology, Odessa 65039, Ukraine

**Keywords:** silymarin, silybin, silibinin, antioxidant, Nrf2, NF-κB, vitagenes, gut

## Abstract

Silymarin (SM), an extract from the *Silybum marianum* (milk thistle) plant containing various flavonolignans (with silybin being the major one), has received a tremendous amount of attention over the last decade as a herbal remedy for liver treatment. In many cases, the antioxidant properties of SM are considered to be responsible for its protective actions. Possible antioxidant mechanisms of SM are evaluated in this review. (1) Direct scavenging free radicals and chelating free Fe and Cu are mainly effective in the gut. (2) Preventing free radical formation by inhibiting specific ROS-producing enzymes, or improving an integrity of mitochondria in stress conditions, are of great importance. (3) Maintaining an optimal redox balance in the cell by activating a range of antioxidant enzymes and non-enzymatic antioxidants, mainly via Nrf2 activation is probably the main driving force of antioxidant (AO)  action of SM. (4) Decreasing inflammatory responses by inhibiting NF-κB pathways is an emerging mechanism of SM protective effects in liver toxicity and various liver diseases. (5) Activating vitagenes, responsible for synthesis of protective molecules, including heat shock proteins (HSPs), thioredoxin and sirtuins and providing additional protection in stress conditions deserves more attention. (6) Affecting the microenvironment of the gut, including SM-bacteria interactions, awaits future investigations. (7) In animal nutrition and disease prevention strategy, SM alone, or in combination with other hepatho-active compounds (carnitine, betaine, vitamin B_12_, *etc.*), might have similar hepatoprotective effects as described in human nutrition.

## 1. Introduction

Silymarin (SM) is a C25 containing flavonoid mixture, extracted from the *Silybum marianum* (milk thistle) plant. Today’s standardized (according to its silibinin, often called silybin, content) SM extract contains approximately 65% to 80% flavonolignans (silybin A and silybin B, isosilybin A, isosilybin B, silychristin and silydianin), with small amounts of flavonoids, and approximately 20% to 35% of fatty acids and polyphenolic compounds possessing a range of metabolic regulatory effects [1]. Silybin was discovered as the first member of a new family of natural compounds called flavonolignans in 1959 [2] and it is known as the predominant and primary active ingredient in SM [3,4]. That is why compounds containing milk thistle ingredients showing silybin content and silybin antioxidant, as well as other activities in various model systems, are used to explain the biological activity of SM. In particular, SM has been the gold standard drug to treat liver disorders of different etiologies and milk thistle extracts have been used as traditional herbal remedies (“liver tonics”) for almost 2000 years. Therefore, SM is most well known for its antioxidant and chemoprotective effects on the liver [5,6,7,8,9,10,11] and it is often prescribed and self-prescribed as a complementary and alternative hepatoprotective medicine [12]. SM is being studied as a hepato-, neuro-, nephro- and cardio-protective ingredient due to its strong antioxidant and tissue regenerative properties [12,13,14,15,16,17]. There is a range of recent comprehensive reviews covering various routes and mechanisms of action of SM in animal models and human trials [13,14,15,16,17] very often referring to its antioxidant properties. However, it seems likely that direct antioxidant (AO) activity of polyphenols does not contribute directly to the antioxidant defence of the body [18,19] and only limited work has been carried out to explore SM/silybin impact on the induction of cellular antioxidant defence via the modulation of various transcription factors, including Nrf2 and NF-κB and respective gene and protein expressions. Potential molecular proliferative signaling targets for anti-cancer activity of silibinin include the receptor tyrosine kinase, STAT, androgen receptor and NF-κB pathways [20], however, anti-cancer activity of SM is beyond the scope of the present review. Therefore, this review focuses on evaluating recent studies on SM (silibinin) antioxidant effects in various *in vitro* and *in vivo* model systems in the context of its contribution to the antioxidant systems regulation and participation in cell signaling.

## 2. Absorption and Metabolism of Silibinin

SM and its main constituent silibinin sources, metabolism and bioavailability have already been reviewed extensively [3,21,22]. It has been shown that after oral consumption silibinin is characterised by comparatively low availability, e.g., in rats it is only about 0.95% [23]. In fact, after the oral administration of the standardized milk thistle extract Legalon, flavonolignans were rapidly absorbed and eliminated [22] with a half-life for silibinin of 6 h [24,25,26]. The main biotransformation route of silybin and derivatives was identified to be conjugation [3]. It is interesting to note that silibinin, similar to other flavonoids is recognized by the body as a foreign matter and quickly metabolized via phase II enzymes. Indeed, oral consumption of silibinin was associated with a significant increase in both glutathione *S*-transferase (GST) and quinone reductase (QR) activities in liver, lung, stomach, skin and small bowel in a dose- and time-dependent manner [27]. Silibinin present in the systemic circulation was found mainly in conjugated form [28,29]. In fact, after oral SM administration to healthy volunteers, only 10% [30] to 17% [31] of the total silibinin in the plasma was found in the free unconjugated form. Indeed, mono-, di-, and sulpho-glucuronides are shown to be formed, and 31 metabolites have been identified [32]. Indeed, silibinin in humans and rats shows fast elimination of both the free and conjugated forms with the mean elimination half-life being 6.32 h [24]. Therefore, similar to other flavonoids, after oral consumption silibinin, the main constituent of SM, is characterised by comparatively low availability, fast metabolism and its concentration in plasma is mainly in nano-molar range and only in some cases reaching micro-molar concentrations.

## 3. Antioxidant Systems of the Body

During evolution, living organisms have developed specific antioxidant protective mechanisms to deal with reactive oxygen species (ROS) and reactive nitrogen species (RNS) [33]. Therefore, it is only the presence of natural antioxidants in living organisms which enable them to survive in an oxygen-rich environment. The general term “antioxidant systems” describes these mechanisms, which are diverse and responsible for the protection of cells from the actions of free radicals. The protective antioxidant compounds are located in organelles, subcellular compartments, or the extracellular space enabling maximum cellular protection to occur. Thus, antioxidant systems of the living cell include three major levels of defence [33,34,35,36,37].

The first level of defence is responsible for prevention of free radical formation by removing precursors of free radicals or by inactivating catalysts and consists of three antioxidant enzymes, namely superoxide dismutase (SOD), glutathione peroxidase (GSH-Px), and catalase (CAT) plus metal-binding proteins. Since the superoxide radical is the main free radical produced in physiological conditions in the cell, SOD (EC 1.15.1.1) is considered to be the main element of the first level of antioxidant defence in the cell. This enzyme dismutates the superoxide radical in the following reaction:

2 O_2_^−^+ 2 H+ → H_2_O_2_ + O_2_

The hydrogen peroxide formed by SOD action can be detoxified by GSH-Px or CAT which reduce it to water as follows:

H_2_O_2_ + 2 GSH → GSSG + 2 H_2_O

2 H_2_O_2_ → 2 H_2_O + O_2_

Transition metal ions also accelerate the decomposition of lipid hydroperoxides into cytotoxic products such as aldehydes, alkoxyl radicals, and peroxyl radicals. Therefore, metal-binding proteins (transferrin, lactoferrin, haptoglobin, hemopexin, metallothionein, ceruloplasmin, ferritin, albumin, myoglobin, *etc.*) also belong to the first level of defence. Unfortunately, the first level of antioxidant defence in the cell is not sufficient to completely prevent free radical formation and some radicals do escape through this level, initiating lipid peroxidation and causing damage to polyunsaturated fatty acids (PUFAs), DNA and proteins. Therefore, the second level of defence consists of chain-breaking antioxidants—vitamin E, ubiquinol, carotenoids, vitamin A, ascorbic acid, uric acid, and some other antioxidants. Glutathione (GSH) and thioredoxin systems also have a substantial role in the second level of antioxidant defence. Chain-breaking antioxidants inhibit peroxidation by keeping the chain length of the propagation reaction as small as possible. Therefore, they prevent the propagation step of lipid peroxidation by scavenging peroxyl radical intermediates in the chain reaction.

However, even the second level of antioxidant defence in the cell is not able to prevent lipid and protein oxidation and some biological molecules are damaged. In this case, the third level of defence is based on systems that eliminate damaged molecules or repair them. This level of antioxidant defence includes lipolitic (lipases), proteolytic (peptidases or proteases) and other enzymes (DNA repair enzymes, ligases, nucleases, polymerases, proteinases, phospholipases, various transferases, *etc.*). All these antioxidants are operating in the body in association with each other forming an integrated antioxidant system. The cooperative interactions between antioxidants in the cell are vital for maximum protection from the deleterious effects of free radicals and toxic products of their metabolism.

Therefore, the antioxidant defences include several options [33,34,35,36,37]:

- Decrease localized oxygen concentration
- Decrease activity of pro-oxidant enzymes and improve efficiency of electron chain in the mitochondria and decreasing electron leakage leading to superoxide production
- Prevention of first-chain initiation by scavenging initial radicals by inducing various transcription factors (e.g., Nrf2 and NF-κB) and ARE-related synthesis of AO enzymes (SOD, GSH-Px, CAT, glutathione reductase (GR), GST, *etc.*)
- Vita-gene activation and synthesis and increased expression of protective molecules (GSH, Thioredoxins, heat shock proteins (HSPs), sirtuins, *etc.*)
- Binding metal ions (metal-binding proteins) and metal chelating
- Decomposition of peroxides by converting them to non-radical, nontoxic products (Se-GSH-Px);
- Chain breaking by scavenging intermediate radicals such as peroxyl and alkoxyl radicals (vitamins E, C, GSH, uric acid, ubiquinol, bilirubin, *etc.*)
- Repair and removal of damaged molecules (methionine sulfoxide reductase (Msr), DNA-repair enzymes, chaperons, *etc.*)

## 4. Antioxidant Properties of Silymarin (SM)

It should be noted that SM can contribute to the antioxidant defences in different ways. Firstly, by direct free radical scavenging. Secondly, by preventing free radical formation by inhibiting specific enzymes responsible for free radical production, or by maintaining the integrity of electron-transport chain of mitochondria in stress conditions. Thirdly, by participating in the maintenance of optimal redox status of the cell by activating a range of antioxidant enzymes and non-enzymatic antioxidants, mainly via transcription factors, including Nrf2 and NF-κB. Finally, by activating an array of vitagenes, responsible for the synthesis of protective molecules, including HSP, thioredoxin (Trx), sirtuins, *etc.*, and providing additional protection in stress conditions. In most studies pure silybin, as the main component of SM, was used, however, in some cases SM also showed antioxidant action *in vivo*.

### 4.1. Direct Free Radical Scavenging

The effects of silibinin on the formation of ROS and eicosanoids by human platelets, white blood and endothelial cells were studied [32]. Silibinin showed to be a strong scavenger of HOCI (IC_50_ 7 µM), but not of O_2_^−^ (IC_50_ > 200 μM) produced by human granulocytes. Similarly, production of O_2_^−^ and NO in isolated rat Kupffer cells were inhibited by silibinin in a dose-dependent manner, with IC_50_ 80 μM [38,39]. Indeed, silybin has no superoxide anion scavenging capability but was able to significantly decrease (at 20 μM) hem-mediated low dencity lipoprotein (LDL) oxidation and showed slight inhibition of hydroxyl radical formation [40]. The rate constants of silybin with OH radical (1.8 × 10^10^ dm/mol/s) is diffusion controlled, suggesting that silybin is a potent free radical scavenger [41]. Indeed, silibinin (2.5 μM) significantly decreased the concentration of H_2_O_2_ in Ab_1–42_-stressed neurons and prevented oxidative-related injuries [42]. Furthermore, silybin (10 μM and higher) is shown to have protective activity in ameliorating DNA damage in a model system [41]. Treatment *in vitro* with silibinin significantly inhibited spontaneous O_2_^−^ and H_2_O_2_ release and TNF-α production by monocytes from pre-eclamptic women. The main effect of silibinin was obtained at 50 μM concentration [43]. Thus, the authors concluded that silibinin exerts anti-oxidative and anti-inflammatory effects on monocytes from pre-eclamptic pregnant women by inhibiting the *in vitro* endogenous release of ROS and TNF-α production.

Antioxidant action of SM (equal to 62 μM of silybin) has been shown in various model systems. For example, IC_50_ for H_2_O_2_ was 38 μM, while IC_50_ for NO was 266 μM [44]. It was shown that SM inhibited 82.7% lipid peroxidation of linoleic acid emulsion; while BHA, BHT, alpha-tocopherol and Trolox indicated inhibition of 83.3, 82.1, 68.1 and 81.3% respectively on peroxidation of linoleic acid emulsion at the same concentration [45]. In another *in vitro* study free radical scavenging activity and antioxidant properties of SM (>200 μM) were showed by four different assays [46]. It is important to mention that the free radical scavenging activity of pure individual compounds of the SM is reported to vary considerably, with silydianin and silychristin being 2-10-fold more active than the silibinin and on a mass basis, SM is shown to be about 8-fold more potent than silibinin as a free radical scavenger [47]. Indeed, SM and silybin (silibinin) are not the same compounds and their AO activities could differ substantially depending on their concentrations in the target tissues. Therefore, in the following sections a distinction will be made between AO activities of SM and its active constituent silybin.

It seems likely, that SM/silibinin AO effects could be on the cells further upstream of the ROS and TNFa release. In fact, SM has been shown to alter trafficking within cells [48,49] and affect the energy status of the cell [50], so the ROS scavenging effects, if any, could be secondary. Furthermore, many of the effects of SM/silibinin described *in vitro* occur at concentrations not currently achievable in humans or animals. Therefore, a direct scavenging ROS by silibinin in biological systems (except the gut) is not likely to substantially contribute to the antioxidant protection.

### 4.2. Protective Effects of Silybin on Mitochondria, a Main Source of Free Radical Production in the Cell

Mitochondria are the primary cellular consumers of oxygen and contain numerous redox enzymes capable of transferring single electrons to oxygen, generating the ROS superoxide (O_2_^−^). It is well documented that mitochondrial enzymes known to generate ROS include the tricarboxylic acid (TCA) cycle enzymes aconitase and α-ketoglutarate dehydrogenase; the electron-transport chain (ETC) complexes I, II and III; pyruvate dehydrogenase and glycerol-3-phosphate dehydrogenase; dihydroorotate dehydrogenase; the monoamine oxidases (MAO) A and B; and cytochrome b5 reductase [51]. Furthermore, mitochondrial insults, including oxidative damage itself, can cause an imbalance between ROS production and removal, resulting in net ROS production. For example, ROS can induce protein modifications, lipid peroxidation and mitochondrial DNA damage, which ultimately results in mitochondrial dysfunction [52]. Many studies have focused on the detrimental effects of ROS, but it is now clear that mitochondrially generated ROS are also involved in regulating intracellular signal transduction pathways that result in cell adaptation to stress [53].

One of the mechanisms responsible for the decrease in oxidative stress is the protective effect of SM/silibinin on mitochondrial structure and function. Indeed SM protects mitochondria from pathological events by triggering pro-survival cell signaling. For example, silibinin supplementation is shown to optimize electron-transport chain, decreasing electron leakage and ROS formation and directly reducing activities of ROS-producing enzymes in the mitochondria. In rats subjected to ischemia/reperfusion (I/R), compared with the control group, a severe impairment of mitochondrial bioenergetics was observed. SM prevented the most significant changes that occurred in mitochondria during I/R (decreased ATP levels, membrane potential and state 3 respiration) and the associated cell dysfunction [54].

Silibinin (100–500 μM), was evaluated for its protective effect against beta-adrenergic agonist isoproterenol-induced injury in cultured rat neonatal cardiac myocytes [55]. It was shown that silibinin addition was associated with increased SOD activity and upregulated mitochondrial membrane potential and with a prevention of mitochondrial dysfunction and cell injury [54]. Silibinin, at a concentration as low as 10 μM, fully mitigated the rise in metabolic flow-driven ROS formation in perfused rat hepatocytes. In addition, studies on isolated liver mitochondria revealed that this low dose of silibinin depressed ROS production linked to the electron transfer chain activity [56]. It has been shown that cold preservation and warm reperfusion of the rat liver were associated with increased lipid peroxidation and superoxide anion generation, as well as with decreased GSH, mitochondrial ATP content and respiratory control ratio (RCR). However, preservation conducted in presence of silibinin (100 μM) improved parameters affected by preservation and reperfusion. Indeed, silibinin promoted an increase of ATP and RCR by 39 and 16% respectively and decreased oxidative stress to values observed in livers never preserved nor perfused [57]. It has been suggested that the uncoupler-like activity of dehydrosilybin could be the basis of its ROS modulation effect in various experimental systems. In fact, dehydrosilybin uncoupled the respiration of isolated rat heart mitochondria with a very high potency in suppressing ROS formation in isolated rat heart mitochondria with IC(50) = 0.15 μM [58]. It is interesting to note that silibinin in mitochondria was far more effective than its effect in a purely chemical system generating superoxide or in cells capable of oxidative burst, where the IC(50) for dehydrosilybin exceeds 50 μM. Changes in mitochondrial respiratory complexes in fatty hepatocytes were also attenuated by silibinin-vitamin E complex (15 mg vitamin E and 47 mg silybin) fed to rats with a major protective effect on Complex II subunit CII-30 [59]. Similarly, silybin (0.4 g/kg) in complex with phospholipid (SILIPHOS) was effective in decreasing severe oxidative stress and preserving hepatic mitochondrial bioenergetics and mitochondrial proton leak and ATP reduction in nonalcoholic steatohepatitis induced by the methionine- and choline-deficient (MCD) diet [60]. Silymarin oil (10 mL/kg/BW) significantly increased levels of membrane fluidity and membrane potential of liver mitochondria [61]. It has been suggested that protective mechanism of action of silibinin (50–200 μM) in intrastriatal MPP+-injected rats may be linked to maintenance of mitochondrial bioenergetics and integrity [62]. An *in vitro* study demonstrated that silibinin inhibits the activity of ROS-generating monoamine oxidase (MAO) that catalyzes the oxidative deamination of monoamines [63]. Similarly, silymarin oil (10 mL/kg/BW) decreased MAO activity in mice livers [61]. The formation of leukotrienes via the 5-lipoxygenase pathway was indicated to be strongly inhibited by silibinin. In particular, in human granulocytes IC50-values of 15 μM and 14.5 μM silibinin were detected for LTB4 and LTC4/D4/E4/F4 formation respectively. However, much higher silibinin concentrations (45–69 μM) were necessary to inhibit the cyclooxygenase pathway [38]. Rats exposed to a carcinogen (1,2-di-methylhydrazine DMH) showed increased activities of phase I enzymes (cytochrome b5, cytochrome b5 reductase, cytochromeP450, cytochromeP450 reductase, cytochromP4502E1) in the liver and colonic mucosa as compared to control rats. Silibinin supplementation (50 mg/kg/BW) modulates the xenobiotic metabolizing enzymes including decreasing activity of ROS-producing cytochrome b5 reductase [64]. Although energy generation in the mitochondrion is an essential and extremely important process for cell survival, excessive mitochondrial ROS production also has detrimental consequences for the cell and the whole body.

The different behaviour of silymarin/silibinin in normal and cancerous cells should be mentioned. In particular, SM is shown to have a protective effect against diabetes-induced cardiomyocyte apoptosis [65] as well as apoptosis caused by various toxicants (see Section 4.4), while it causes apoptosis in cancerous cells. For example, SM effectively suppressed cell growth in a dose- and time-dependent manner, and arrested cell cycle progression at G1/S phase in human ovarian cancer line A2780s and PA-1 cells via up-regulation of p53, p21, and p27 protein expression, and down-regulation of CDK2 protein expression [66]. Furthermore, in the aforementioned study the silymarin treatment significantly induced apoptosis in A2780s and PA-1 cells by increasing Bax and decreasing Bcl-2 protein expression, and activation of caspase-9 and caspase-3. Indeed, anti-cancer properties of SM are well characterised but they are beyond the scope of the review.

### 4.3. Inhibition of Free-Radical Producing Enzymes by Silybin

#### 4.3.1. Xanthine Oxidase

Xanthine oxidase (XO) catalyzes the terminal two steps of purine degradation, converting hypoxanthine to uric acid and is considered to be a critical source of both O_2_^−^ and H_2_O_2_ in inflammatory diseases [67]. In particular, XO in its oxidase form is considered to be a main source of oxidative stress and destructive free radicals in ischemia reperfusion injury associated with heart attacks, stroke and spinal cord injury, as well as being a destructive force in myocardial or renal hypoxia and infarctions [68]. It seems likely that silibinin can affect XO activity, and inhibition of XO by silibinin in the steady-state appears to be independent of the silibinin concentration. Furthermore, in the presence of 50 μM silibinin, the production of superoxide by XO was decreased by approximately 20% relative to the control at 100 μM xanthine [69]. When O_2_^−^ production was induced in xanthine/xanthine oxidase system, silybin inhibited uric acid formation with an IC_50_ of 32.2 μM [68]. Indeed, inhibition in uric acid formation occurred parallel with inhibition of cytochrome C reduction (e.g., with O_2_^−^ formation) suggesting that the observed effects are due to the inhibition of xanthine oxidase activity rather than the scavenging of O_2_^−^ radicals.

#### 4.3.2. NADPH Oxidase

ROS generation by NADPH oxidase enzyme complex plays a critical role in several physiological processes including host defense, posttranslational modification of proteins, cell differentiation and regulation of gene expression [70]. Silybin and its derivatives inhibited the NADPH oxidase activity in PMA-stimulated cell lysate in a concentration dependent manner. The most distinct inhibition was observed when cell lysate was pre-incubated with 5,7,4′′-trimethylsilybin possessing high lipid solubility. Indeed, 50% inhibition of NADPH oxidase activity was achieved when using 5 μM of 5,7,4′′-trimethylsilybin and 10 μM of silybin at final concentrations [68]. It is interesting to note that As-intoxicated rats showed a significant (*p* < 0.05) up-regulation of myocardial NADPH (NOX) oxidase sub units such as NOX2 and NOX4 as well as Keap-1, and down-regulation of Nrf2 and heme oxygenase (HO-1) protein expressions. Pre-administration of silibinin (75 mg/kg/BW) remarkably recovered all those altered parameters to near normalcy in As-induced cardiotoxic rat [71]. Co-administration of silibinin (75 mg/kg/BW) along with As-resulted in a reversal of As-induced biochemical changes in the kidney, accompanied by a significant decrease in lipid peroxidation and an increase in the level of renal antioxidant defense system [72]. It also decreased the NADPH oxidase, iNOS and NF-κB over expression by As and upregulated the Nrf2 expression in the renal tissue. The effect of silybin in cultures of mouse podocytes and in the OVE26 mouse, a model of type 1 diabetes mellitus and diabetic nephropathy was investigated. Exposure of podocytes to high glucose increased intracellular superoxide production by 60%, NADPH oxidase activity by 90%, NOX4 expression by 100%, and the number of apoptotic cells by 150%, effects that were completely blocked by 10 μM silybin. These *in vitro* observations were confirmed by similar *in vivo* findings. The kidney cortex of vehicle-treated control OVE26 mice displayed greater NOX4 expression and twice as much superoxide production than cortex of silybin-treated mice [73]. Therefore, the *in vitro* data demonstrated that silybin decreased superoxide generation in cultured podocytes and the *in vivo* study showed a similar effect in the kidney cortex. This is not surprising considering that oxidative stress and enhanced generation of ROS is the underlying mechanism of injury to multiple cell types.

### 4.4. Antioxidant Protective Properties of SM (Silibinin) in Prevention of Toxic Effects of Various Chemicals

The liver is a primary organ involved in biotransformation of food toxicants and drugs. Toxins absorbed from the intestinal tract first enter the liver resulting in a variety of liver disorders. Hepatic disorders are mainly caused by toxic chemicals (alcohol), xenobiotics (carbon tetrachloride, chlorinated hydrocarbons), anticancer (doxorubicin, cisplatin), immunosuppressant (cyclosporine), analgesic anti-inflammatory (paracetamol, thioacetamide), anti-tubercular (isoniazid, rifampicin) drugs, radiations (gamma radiations), heavy metals (cadmium, arsenic), mycotoxins (aflatoxin, T-2 toxin, fumonisins, ochratoxins), galactosamine, lipopolysaccharides, *etc.* [74]. Increasing evidence supports the important role of oxidative stress as a key mechanism of hepatotoxicity. Therefore, the aforementioned toxicants were used in various experimental models to assess antioxidant protective properties of SM. These are especially important, since SM is primarily used in clinical practice to protect, support and repair the liver.

#### 4.4.1. Arsenic

As-induced toxicity is associated with increased production of ROS, elevation in lipid peroxidation, protein carbonylation and DNA oxidation, and disruption of the cell cycle that results in apoptosis [75]. Indeed, the toxic effect of arsenic was indicated by significantly decreased activities of AO enzymes (SOD, CAT, GSH-Px, GST, GR and glucose 6-phosphate dehydrogenase (G6PDH)) along with non-enzymatic antioxidants (GSH, vitamins C and E) in the rat liver; [76] and heart [71] and decreased Nrf2 and HO-1 activities in the heart [71]. Similar As-induced changes were seen in the rat kidney, including decreased activity of AO enzymes (SOD, CAT, GSH-Px, GSH, *etc.*), GSH, ascorbic acid and vitamin E, increased thiobarbituric acid reactive substances (TBARS), lipid peroxides and protein carbonyls, overexpression of NADPH oxidase, iNOS and NF-κB [72]. Silibinin was effective in counteracting all the parameters of oxidative stress. Indeed, administration of silibinin (50–75 mg/kg/BW) exhibited a significant preservation of antioxidant defences by maintaining activity of AO enzymes and non-enzymatic antioxidants in As-treated rats [76,77,78]. All the changes were supported by reduction of DNA damage in hepatocytes and histopathological observations of the liver.

#### 4.4.2. Carbone Tetrachloride

Carbone tetrachloride (CCl4) is considered as an important hepatotoxin due to its severe oxidative effect on the liver. Indeed, metabolism of CCl4 via CYP2E1 to highly reactive ROS plays a major role in the mode of action of the toxicant [79]. It is well established that CCl4 inhibits AO enzymes (SOD, GSH-Px and CAT) and GSH in liver samples [80], increases the secretion of ALT, aspartate transaminase (AST) and ALP due to hepatic injuries caused by ROS [81] and enhances TBARS in the liver [82] and serum [83]. It has been shown that practically all elevated indexes of the oxidative stress caused by CCl4 were restored almost to the initial physiological levels by SM treatment (25–100 mg/kg/BW) [80,81,82,83,84,85,86,87] and hepatic injuries were significantly decreased. It is important to mention that in a model system based on CCl4-induced liver fibrosis in mice, microarray analysis showed that SM downregulated the expression levels of cytoskeleton organization genes and mitochondrion electron-transfer chain genes, such as cytochrome c oxidase, Cox6a2, Cox7a1, and Cox8b genes [88]. Furthermore, SM (0.1–1.0 g/kg diet) had a protective effect against CCl4-induced liver damage and AO system defences in common carp [89].

#### 4.4.3. Mycotoxins

It has been shown that major mycotoxins (aflatoxins, AF; ochratoxin A, OTA; T-2 toxin and fumonisins, FM) cause oxidative stress and apoptosis in the cell [90,91,92]. Indeed, mycotoxins generate ROS, which induce lipid and protein oxidation leading to changes in membrane integrity, cellular redox signaling, and in the antioxidant status of the cells. Silibinin is shown to be a potent protective compound against apoptosis and cytotoxicity caused by OTA [93]. In fact, silibinin in doses from 130 to 260 μM prevented chromatin condensation, caspase-3 activation, apoptotic DNA fragmentation and lipid peroxidation that were induced by OTA, H_2_O_2_ and ActD/TNF-α, respectively. Furthermore, silibinin (0.04–26.0 μM) had hepatoprotective effects against OTA- or LPS-mediated TNF-alpha release and also reduced the cytotoxicity of both compounds [94]. Pre-treatment of the rats with SM (100 mg/kg/BW) prior to AFB1 was found to show significant protection against AFB1-induced liver damage, as evidenced by a significant lowering of the activity of the serum enzymes glutamic-oxaloacetic transaminase (GOT) and glutamic-pyruvic transaminase (GPT) and enhanced hepatic reduced GSH status [95]. SM (100 mg/kg/BW) also ameliorated toxic liver damage caused by FB₁ in BALB/c mice by preventing elevation of the levels of caspase-8 and tumor necrosis factor-alpha mediators [96].

#### 4.4.4. Thioacetamide

Thiacetamide (TAA) is a potent hepatotoxicant which requires metabolic activation by the mixed-function oxidases. In the development of TAA-induced toxicity, free radicals are thought to play a critical role [97] and TAA-induced cirrhosis resulted in oxidative stress in the liver, as observed by a high level of lipid peroxidation, accompanied by distorted status of antioxidants [98]. SM is shown to have a protective effect against oxidative stress caused by TAA in rats. Liver fibrosis was induced in male Sprague Dawley rats by TAA administration (0.03% w/v) in drinking water for a period of 12 weeks. SM (50 mg/kg/BW) was shown to significantly increase hepatic antioxidant enzymes (SOD, CAT and GSH-Px) activity in the TAA-treated rats [99]. Indeed, a significant decrease in TAA-induced liver damage was observed in SM-pre-treated rats indicated by a reduction in serum GPT, GOT and alkaline phosphatase (ALP), restoration of the antioxidant system (SOD, CAT, GR, GSH-Px and GST) and decreased lipid peroxidation [100]. Increases in hepatic levels of malondialdehyde (MDA) and protein carbonyls in rats associated with TAA administration were partially blocked by SM consumption [101]. Furthermore, GSH depletion and heat shock protein-47 gene expressions were also decreased in response to SM administration.

#### 4.4.5. Cisplatin

Cisplatin (CDDP) is a chemotherapeutic drug widely used against a variety of cancers and its nephrotoxicity is mainly due to ROS production and oxidative stress [102]. It was shown that CDDP caused decreased activities of AO enzymes (SOD and GSH-Px) and GSH, increased MDA in rat liver [103,104] and significantly elevated serum activities of lactate dehydrogenase (LDH) and creatine kinase (CK) [104]. SM (100 mg/kg/BW) significantly prevented the cisplatin-evoked disturbances in the above-mentioned antioxidant indexes [103,104]. Furthermore, *in vitro* pre-treatment with 25–200 μM of SM significantly protected against cisplatin-induced cell death in a dose-dependent manner [105], inhibited apoptotic cascade and increased cell viability in the HEI-OC1 cells [106].

#### 4.4.6. Mn Toxicity

It is well known that manganese toxicity in animals is associated with increased oxidative stress, apoptosis and inflammation [107]. In fact, animals exposed to manganese chloride were characterised by a significant increase in TBARS levels associated with a decrease of enzymatic (SOD, CAT, GSH-Px) and non-enzymatic (GSH, Vitamin C) antioxidants [108,109], increased lipid and protein oxidation [110], DNA fragmentation and urinary hydrogen peroxide [111]. Co-administration of SM (100 mg/kg/BW) to Mn-treated rats significantly restored antioxidant defences and attenuated oxidative damages observed in the liver, kidney and brain [108,109,110,111].

#### 4.4.7. Benzo[a]pyrene

In experiments with benzo(a)pyrene (B(a)P), an environmental contaminant causing oxidative stress, the protective effect of SM/silibinin (400–500 μM) was shown. It was associated with decreasing DNA damage and apoptosis [112], preventing protein thiol oxidation and AO enzyme (SOD, GSH-Px, CAT, GR, GST) inhibition in the hemolysate [113], restoring redox status, modulating glutathione metabolizing enzymes, decreasing formation of protein oxidation products and changing the level of antioxidant enzymes (5 mM; [114]). SM (50–250 mg/kg/BW) showed substantial protective effects against B(a)P-induced damages by inhibiting phase I detoxification enzyme CYP1A1 and modulating phase II conjugating enzymes (glutathione-*S*-transferase, epoxide hydroxylases, uridine diphosphate glucuronosyltransferases, NAD(P)H: quinone oxidoreductase 1, sulfotransferases) in rat liver [115].

#### 4.4.8. Doxorubicin

Doxorubicin (DOX, also called adriamycin) is an effective chemotherapeutic agent.However, its side effect is cardiotoxicity, caused by oxidative stress, mitochondrial dysfunction and apoptosis [116]. It has been shown that DOX-induced biochemical and histopathological alterations in rat testis could be prevented and/or protected by SM (50 mg/kg/BW). The SM protective and preventive effects which result in the reduction of DOX-induced carbonyl stress and DNA damage [117], decrease in the plasma creatine phosphokinase (CPK), LDH, creatinine and urea, myocardial MDA contents and renal tissue contents of MDA and GSH have also been shown [118]. Similarly, SM (16 mg/kg/BW) significantly decreased an oxidative stress in DOX-treated rats by decreasing MDA and DNA fragmentation in the liver [119]. A protective influence of SM (60 mg/kg/BW) on the heart and liver tissue against toxicity induced by DOX in rats was associated with a prevention of an increase in AST and CK serum activity [120].

#### 4.4.9. Ethanol

Oxidative stress plays an important role in the pathogenesis of alcoholic liver damage. During ethanol metabolism, ROS and RNS are formed causing oxidative stress [121]. Supplementation with a standardized SM (200 mg/kg/BW) attenuated an oxidative stress caused by acute ethanol administration, including prevention of elevation of serum ALT activity, decrease in hepatic GSH and enhanced lipid peroxidation, and increased hepatic TNF production [122]. Similarly, SM (100–200 mg/kg/BW) effectively protected liver from alcohol-induced oxidative stress as evidenced by reducing ALT and AST activities in serum, increasing SOD and GSH-Px activities and decreasing MDA content in the liver [123]. Furthermore silybin (250 mg/kg/BW) normalized alcohol-altered AO parameters in the whole blood hemolysate of mice, including AO enzymes (SOD, CAT, GR, GSH-Px), GSH and TBARS [124].

#### 4.4.10. Other Toxicants

The protective effects of similar doses of SM/silybin on the antioxidant systems of the body are also shown in pyrogallol toxicity [125,126,127], bleomicyn-injected mice [128], acrolein toxicity in mice [129], diethylnitrosamine-induced oxidative stress in rat liver [130], sodium fluoride [131] and sodium nitrite [132] toxicity.

Indeed, in animal experiments and *in vitro* studies SM/silibinin blocked the oxidative toxicities of As, CCl4, various mycotoxins, thioacetamide, cisplatin, Mn overload, benzo[a]pyrene, doxorubicin, ethanol and some other toxicants by decreasing an abnormally high production of ROS in living cells and restoring signal transduction and gene expression. In the aforementioned studies the free radical scavenging and antioxidant properties of SM and silibinin are demonstrated by: (a) restoration of the endogenous AO enzymes (SOD, CAT, GSH-Px, GR and GST) and non-enzymatic antioxidants (vitamins E and C) in the liver and other tissues of stressed animals; (b) increased intracellular concentration of GSH in liver and other tissues; (c) decreased lipid and protein oxidation, detected as reduced MDA/TBARS and carbonyl content; (d) decreased DNA fragmentation/damage and apoptosis; (e) reduced secretion of ALT, AST, ALP from the liver into the plasma due to hepatic injuries caused by ROS; (f) restored Nrf2 and HO-1 activities; (g) reduced NF-κB expression and concentration of pro-inflammatory cytokines, including tumor necrosis factor. It seems likely that the use of SM before or soon after a toxic insult is more protective than in chronic liver disease [133]. A number of *in vitro* and *in vivo* studies have also shown an anticancer protective effect of SM and silibinin in various *in vitro* and *in vivo* model systems and they were comprehensively reviewed [124,125,126,127,128,129,130,131,132,133,134,135,136,137,138]. However, because of limitation of space in this review they will not be included in the analysis.

### 4.5. Is Effective Silybin Concentration in Vitro Achievable in Vivo?

As mentioned above, because of their poor absorption and rapid elimination, plasma values of silybin will rarely exceed micromole concentrations. Therefore, the SM concentration used in most of the aforementioned studies are not achievable in the *in vivo* target tissues, where the silybin concentration is usually at least 10-100-fold lower. Indeed, the active free silibinin concentration in plasma after oral consumption of SM, depending on dose of supplementation, could be in the range 0.2–2.0 μM. For example, in healthy volunteers, after an oral administration of SM (equivalent to 120 mg silibinin), total (unconjugated + conjugated) silibinin concentration in plasma was 1.1–1.3 μg/mL [139]. Lower bioavailability of silibinin was shown in another study with higher dose of SM consumption (equivalent to 240 mg silibinin), where maximum silibinin concentration was about 0.7 μM [24]. Comparable silibinin concentrations (0.3–1.9 μM) were reported in healthy volunteers receiving about 240 mg silibinin [140]. More recently, silybin A maximal concentration in human plasma was shown to be 0.2–0.6 μM and silybin B concentrations comprised about 30% of those shown for silybin A [22]. The concentrations of free and conjugated silibinin in the target tissues of mice have been shown to reach their maximum levels within 1 h after 50 mg/kg/BW silibinin administration. The *C*_max_ of free/conjugated silibinin in the tissues were 18.3/11.8; 8.9/5.8; 255/560; 2.9/8.9; 5.3/12.6; 12.0/21.9 μM for liver, lung, stomach, skin, prostate and pancreas, respectively [27]. Indeed, the average concentration of most plant polyphenols in plasma rarely exceeds 1–2 μM in healthy subjects [141,142,143]. Furthermore, the effect of SM in physiologically relevant concentrations would be relatively limited when taking into account concentrations of other antioxidants in plasma. For example, vitamin C (26.1 to 84.6 μM), vitamin E (20–30 μM), urate (150–200 μM), and albumin (several hundred μM) are found in plasma [19,144] and polyphenol contribution to plasma TAC will certainly be less than 2% [19]. A similar situation could be found in animals (chickens) where antioxidant concentrations in plasma are in the same range—vitamin C: 61–66 μM; [145,146,147]; vitamin E: 13–15 μM [34]; and total antioxidant value in plasma could vary from 336.9 μM [147] up to 740–830 μM, [148]. Indeed, the general concentration of antioxidants in human plasma is about 1000 μM [149], therefore 20–50 μM additional antioxidant from dietary sources would be required to make a significant contribution to systemic antioxidant capacity [150]. While SM displays potent antioxidant activity *in vitro*, the bioactive forms of flavonoids *in vivo* are not those forms found in plants (*i.e.*, flavonoid glycosides) due to their extensive biotransformation in the small intestine and hepatic metabolism upon absorption. Therefore, metabolic modifications occurring *in vivo* may substantially influence the antioxidant activity of dietary flavonoids. Silibinin, similar to other flavonoids, is recognized by the body as foreign matter and quickly metabolized via phase II enzymes.

The aforementioned data clearly indicates that SM protects cells against the damaging effects of oxidative/nitrosative stress and inflammation. It also prevents unwanted cell proliferation in cancer cells [66] as well as pathogen infection [151].

## 5. Oxidative Stress and Transcription Factors

Oxidation-reduction (redox) based regulation of gene expression appears to be a fundamental regulatory mechanism in cell biology. Indeed, it is well established that the level of ROS in the body is rigidly controlled by the antioxidant systems and an excess of free radical production and severe oxidative stress often leads to widespread oxidative damage and cell death. However, a basal level of oxidative stress is essential for cell survival. In fact, a moderate level of oxidative stress, induced by a variety of stressors, can create adaptive responses and improve adaptive ability to stressful challenges [152]. Therefore, a concept of the cellular antioxidant defence has recently been revised with special attention paid to cell signaling. Indeed, in animals redox-signaling pathways use ROS to transfer signals from different sources to the nucleus to regulate a number of various functions including growth, differentiation, proliferation and apoptosis. Various transcription factors are involved in a regulation of the antioxidant defence system [153,154,155,156]. These pathways operate in a coordinated manner and several are critically important for animals to cope with oxidative stress insults. They include Keap1/Nrf2, NF-κB, Mapk and AP-1 [157]. In particular great attention has been paid to a basic leucine zipper transcription factor, Nuclear factor-erythroid-2- (NF-E2-) related factor 2 (Nrf2).

### 5.1. Transcription Factor Nrf2

The recent information on Nrf2 functions can be summarized as follows. Based on the existing evidence, Nrf2 is considered to be the redox-sensitive master regulator of oxidative stress signaling. Growing evidence has demonstrated that the Nrf2 antioxidant response pathway plays an important role in the cellular antioxidant defense by activating a wide variety of genes involved in early defence reactions of higher organisms [158,159]. Indeed, Nrf2 is considered to have a significant role in adaptive responses to oxidative stress, being responsible for the induction of the expression of various antioxidants to combat oxidative and electrophilic stress [160]. In particular, critical components of the cellular antioxidant defense mechanisms include the ROS scavengers, phase II detoxification enzymes, and other detoxification proteins, which contain antioxidant response elements (AREs) in their promoter regions [161,162,163]. There is considerable experimental evidence suggesting that under normal physiological conditions, Nrf2 is kept in the cytoplasm by forming an inactive complex with the negative regulator, Kelch-like-ECH-associated protein 1 (Keap1), which is anchored to the actin cytoskeleton. In fact, cytosolic protein Keap1 sequesters Nrf2 in the cytoplasm and forwards it to a Cul3-based E3 ligase with the following rapid ubiquitin-proteasome degradation resulting in a short (about 20 min) half-life of Nrf2 under normal conditions (for review see [164]. Therefore, Keap1 acts as a redox sensor and upon exposure to oxidative or electrophilic stress, critical cysteine thiols of Keap1 are modified and Keap1 loses its ability to ubiquitinate Nrf2. Furthermore, phosphorylation of Nrf2 at specific serine and/or tyrosine residues also causes an Nfr2-Keap1 dissociation resulting in Nrf2 release and translocation to the nucleus. In the nucleus, Nrf2 combines with a small musculoaponeurotic fibrosarcoma protein called Maf to form a heterodimer [165]. Indeed, Nrf2 binds to the ARE in the upstream promoter region of genes encoding various antioxidant molecules, leading to the expression of antioxidant proteins, thiol molecules and other protective molecules. This includes enzymes of the first line of the antioxidant defence, namely SOD, GSH-Px and CAT, as well as detoxification enzymes (HO-1, NQO1, GST), GSH-related proteins (γ-GCS), NADPH-producing enzymes and others stress-response proteins contributing to counteracting oxidative and inflammatory damage [166,167]. The aforementioned proteins are vital to the maintenance and responsiveness of a cell antioxidant system. Therefore, an orchestrated change in gene expression via Nrf2 and the ARE is responsible for a synergistic protective effect against oxidative stress [168]. There is considerable experimental evidence suggesting that Nrf2 is controlled through a complex transcriptional/epigenetic and post-translational network that ensures its activity increases in response to redox disturbances, inflammation, growth factor stimulation and nutrient/energy fluxes, orchestrating adaptive responses to diverse forms of stress [169]. Nrf2 can be activated by various mechanisms, including stabilization of Nrf2 via Keap1 cysteine thiol modification and phosphorylation of Nrf2 by upstream kinases [170,171]. Beyond the activation of synthesis of antioxidant molecules, Nrf2 also contributes to adaptation by up-regulating the repair and degradation of damaged macromolecules, and by modulating intermediary metabolism conducting metabolic reprogramming during stress [167]. It is important to note that the expression of Nrf2 has been shown throughout human tissue, with high expression in detoxification organs, including the liver and kidney [160]. It has been suggested that low intensity oxidative stress is mainly sensed by Keap1/Nrf2 system with the following downstream up-regulation of the protective AO genes. Intermediate oxidative stress also increases the activity of antioxidant enzymes, but mainly via NF-κB and AP-1 pathways. At both low and intermediate intensity oxidative stresses, MAP-kinases and other kinases are also involved in signal sensing and cellular response, leading to enhanced antioxidant potential [157]. Emerging evidence also indicates that Nrf2 actively interacts with other transcription factors, including heat shock factor (Hsf1; [172]) providing additional options for AO system regulation.

It has been shown that naturally occurring triterpenoids affect Nrf2/ARE pathway and its downstream targets by triterpenoids were shown to be protective in different diseases [173,174,175]. Furthermore, Nrf2 activation and subsequent regulation of the transcription of the plethora of protective genes may be increased due to the chemical modifications of the triterpenoid structure [173].

#### 5.1.1. SM and Nrf2 Regulation

An interesting approach to explain health-promoting properties of polyphenols could be to explore the modulation of signaling pathways, e.g., the Nrf2/Keap1/ARE and NF-κB pathways, resulting in increased expression of genes encoding for cytoprotective molecules, including AO enzymes and phase II detoxification enzymes. In addition, decreased expression of NF-κB-regulated genes would reduce production of pro-inflammatory cytokines. Indeed, it has been shown that some phytochemicals (sulforaphane, curcumin, EGCG, diallyl sulfide, resveratrol, *etc.*) at concentrations as low as 10 μM can activate Nrf2 signaling by inducing phosphorylation of Nrf2 via activation of upstream protein kinases and/or through direct interaction with Keap1 cysteine thiols [170]. It is interesting to note that anthocyanins activate the antioxidant transcription factor Nrf2, at a concentration as low as 1 μM [123]. Silymarin’s ability to upregulate the master antioxidant coordinator Nrf2 has also been investigated in various *in vitro* and *in vivo* model systems. Initially, the SM-containing supplement Protandim (ashwagandha, bacopa extract, green tea extract, SM, and curcumin) was shown to induce HO-1 in a cell culture primarily through Nrf2 activation [176]. Protandim also induces Nrf2 nuclear localization and antioxidant enzyme expression [177]. However, it is difficult to distinguish the SM effect in the mixture. Furthermore, it was shown that SM (a water soluble formulation Legalon-SIL containing a succinate-modified active compounds) at 50 and 100 μM significantly upregulated Nrf2 protein levels in CON1 cells after 48h treatment [178]. SM (104 μM) protected human cells against the damage from the herbicide paraquat via triggering anti-oxidant related genes, including Nrf2, NQO1 and HO-1 [179]. In an *in vitro* experiment with human hepatic HepG2 cells, SM (100 μM) was shown to be protective against AAPH-induced oxidative stress and apoptosis and the antioxidant potential of SM was correlated with induction of antioxidant genes including HO-1, NQO-1, γ-GCLC and SOD via transcriptional activation of Nrf2 [180].

In a dietary rat model of non-alcoholic steatohepatitis there was an increase in nuclear translocation of Nrf2 protein in SM-fed animals [181] which could enhance the protective effect against oxidative stress. Numerically, there was also a trend in increasing Nrf2 endogenous expression protein in rat liver tissue damaged by CCl4 by pre-treatment with SM (100 mg/kg/BW [182]) as well as in an alcohol-pyrazole-fed rat model (50 mg/kg/BW [183]). It seems likely that the effect of SM on the Nrf2 activation in various oxidative-stress related animal models depends on the dose of SM used. For example, at 10 mg/kg/BW SM showed protective effects against dimerumic acid-induced changes in GSH and HO-1 levels, but did not change Nrf2 levels [166]. Indeed, SB-treatment (75 mg/kg/BW) ameliorated arsenic-induced nephrotoxicity by abrogation of oxidative stress, inflammation and apoptosis in rats [72]. It is interesting to note that beyond the preventive action of SB, in rats fed silibinin alone there was a significant increase in Nrf2 mRNA levels in the kidney. The same SB-treated rats were characterised by increased antioxidant defences as indicated by increased renal SOD and GSH-Px activities, as well as enhanced levels of GSH, vitamins E and C. In fact, SB-induced activation of Nrf2 and HO-1 played a significant role in cellular defence against As-induced oxidative cardiac damage. In a recent study, co-treatment with SM (86 mg/kg/BW) significantly enhanced the antioxidant defence systems of the ethanol-consuming mice via the activation of Nrf2 and HO-1 expression in damaged livers [184]. Furthermore, SM (200 mg/kg/BW) significantly up-regulated HO-1 and Nrf-2 in the liver of AAPH-treated mice [180].

Therefore, it is clear from the aforementioned data that SM/silibinin, similar to other polyphenols, acts as an indirect antioxidant through its ability to induce Nrf2 transactivation, which is responsible for the synthesis of an array of antioxidant molecules decreasing oxidative stress and providing effective protection in various stress conditions. This changes an attitude to SM, a compound that has been assumed to act typically as a free radical scavenger and traditional antioxidant. It is still not clear if physiologically-relevant (up to 2 μM) silybin concentrations would upregulate Nrf2, but *in vivo* results obtained with various model systems clearly indicated an activation of Nrf2 by silibinin. In general, most of the experimental studies suggest that dietary SM/silibinin activates antioxidant pathways such as Nrf2/HO1 and downregulates NFκB (see Section 5.2.1. below), MMPs [44,101,185], PPAR [186,187], HIF-1 [188,189,190] and STAT [191,192,193] pathways.

#### 5.1.2. Oxidability and Pro-Oxidant Properties of SM

It has been shown that the oxidation of Keap1 cysteine thiols can be mediated by some polyphenols. It is an interesting fact that among flavonoids, the higher their intrinsic potential to generate oxidative stress and redox cycling, the stronger their potency as inducers of ARE-mediated gene expression [194]. It was hypothesized that low concentrations of polyphenols could generate H_2_O_2_ and activates Nrf2 signaling, inducing cell adaptation to oxidative stress [195]. Thus, polyphenols act as nutritional “medicines”, which might have a preventative nature, rather than functioning as therapeutic agents. Therefore, the activation of Nrf2-ARE signaling by antioxidant polyphenols to induce various AO molecules is probably attributable to their prooxidant activity [170]. It seems likely that the same can be applied for silybin. In fact, pure silybin was found to be unstable whilst silybin in SM was stable in buffers from pH 1.0 to 7.8. The metabolism of silybin was more severe in its pure form compared to silybin in SM, as tested in a range of biological fluids including plasma, intestinal fluid and liver homogenates. It would appear that components in SM have a stabilization effect on its main component silybin [196]. Therefore, it could well be that in some *in vitro* experiments with cell cultures, silibinin could be oxidised and its effects would be a reflection of the oxidised form of the flavonoid or ROS generated during silybin oxidation. For example, silybin and 2,3-dehydrosilybin were shown to chelate transition metals, especially Cu^2+^ and pro-oxidant properties of such a complex *in vitro* have been shown [197]. Similarly, silybin can cause pro-oxidant effects via iron-catalyzed oxidation with the subsequent generation of reactive oxygen species [198]. Silibinin has been shown to be a strong pro-oxidative agent, *i.e.*, it was able to oxidize NADH *in vitro* in the presence of peroxidase and H_2_O_2_ [50,199]. This pro-oxidative action results from the production of free-radical derivatives and subsequent NADH oxidation [200,201]. Treatment with SM (50, 100, or 200 μM) for 24 h affected the cellular redox status and induced a dose-dependent increase in ROS generation in HepG2 cells. There was also a dose-dependent decrease in intracellular GSH level and decreased total AO potential in HepG2 cells [202]. Silibinin induced cell death in human breast cancer cell lines MCF7 and MDA-MB-231 and it was attenuated by antioxidants, suggesting that the effect of silibinin was dependent on ROS generation [203]. Similarly, treatment of HT29 cells with silibinin increased the intracellular ROS level [204]. It has been suggested that SM at 100 mg/kg/BW without stressors might exert pro-oxidant effects in animals. In fact, the levels of NO and MDA increased in CCl_4_ and SM (100 mg/kg/BW)-treated groups, while SM at lower dose levels (25 and 50 mg/kg/BW) did not alter the NO and MDA content in the rat hippocampus. The concentration of total thiol molecules increased in the SM50 group and showed a remarkable decrease in CCl_4_ and SM100 groups [117]. From the aforementioned results, we can suggest that silybin can affect Nrf2 activity via its oxidation-reduction potential.

### 5.2. Transcription Factor NF-κB

The nuclear factor-kappa B (NF-κB) is a widely expressed, inducible transcription factor that has been implicated in regulation of many cellular processes, including inflammation. NF-κB, consisting of a group of five related proteins that are capable of binding to DNA, is activated by a wide range of stimuli including oxidative stress. It regulates the transcription of a range of genes, including pro-inflammatory cytokines and leukocyte adhesion molecules, acute phase proteins and anti-microbial peptides [205,206,207]. Similar to Nrf2, in normal physiological conditions, NF-κB is found in cytoplasm in an inactive state associated with the inhibitory IκB proteins preventing its binding to target sites. Activation of NF-κB is considered to be an effective mechanism of host defense against infection and stress [208]. Indeed, in response to stimuli, including cytokines and other stressors, IκB proteins are rapidly phosphorylated by IκB kinase on specific serine residues, with following ubiquitination, and degradation by the 26S proteasome. The resulting release of NF-κB and subsequent translocation to the nucleus orchestrates the transcription of target genes, responsible for cell survival and involved with inflammation, immunity, apoptosis, cell proliferation and differentiation [209].

There are also other stimuli implicated in NF-κB activation including cell-surface receptors, inhibitory κB kinases, IκB proteins and factors that regulate the posttranslational modification of the Rel proteins, *etc.* [206,207,208,209]. Current information indicates that there are complex interplay/crosstalk mechanisms between Nrf2 and NF-κB pathways. On the one hand, NF-κB pathway is inhibited by several Nrf2 activators. On the other, NF-κB may directly antagonize the transcriptional activity of Nrf2 (for review see [206]). In recent years, several compounds, including various polyphenols, have been isolated from plants that have inhibitory activities against multiple components of NF-*κ*B activation pathway and it seems likely that SM could also have inhibitory activity on NF-κB.

#### 5.2.1. SM and NF-κB Regulation

The anti-inflammatory activity of phenolic compounds has been demonstrated in a number of *in vitro* and *in vivo* studies and polyphenols may affect inflammation mainly as modulators of inflammatory redox signaling pathways [210]. The polyphenolic compounds express anti-inflammatory activity by modulating the expression of pro-inflammatory genes such as cyclooxygenase, lipoxygenase, nitric oxide synthases and several important cytokines, mainly acting through nuclear factor-κB and mitogen-activated protein kinase signaling [210,211]. Due to the large number of studies that have demonstrated regulatory effects of SM/silybin on the expression of NF-κB in various *in vitro* and *in vivo* models there is insufficient space in this review to analyze all of them, so we will focus only on recent investigations addressing the issue. It is well established that various plant-derived polyphenols can suppress TNF-α activated, NF-κB-associated inflammatory pathways both *in vitro* and *in vivo*. These polyphenols include SM as well as curcumin, resveratrol, genistein, epigallocatechin gallate, flavopiridol, emodin, morin isoliquiritigenin, naringenin, ellagic acid, apigenin, kaempferol, catechins, myricetin, xanthohumol, fisetin, vitexin, escin, mangostin and others [210]. SM has long been known to inhibit the activation of inflammatory NF-κB signaling [212]. Recent studies have confirmed that the protective anti-inflammatory effects of SM/silybin could be mediated by their inhibitory potential on the NF-κB, which is a key transcriptional factor for numerous genes involved in regulation of inflammation, immune system, cell differentiation, survival, apoptosis, *etc.* Here, we give some examples of participation of SM/silibinin in NF-κB pathway inhibition.

##### 5.2.1.1. *In Vitro* Studies

Silybin is a potent inhibitor of NF-κB activation. Indeed, Manna *et al*. [213] tested silybin in a number of *in vitro* human cell experimental systems and found it inhibited TNF-mediated NF-κB activation in a dose-dependent manner with maximum inhibition at 50 μM and it regulated NF-κB 100 times more effectively than aspirin. Furthermore, silibinin (80 μM) protected glial cells against peroxide-induced ROS formation, ATP depletion, and cell damage [214]. Interestingly, the inhibition of peroxide-induced ROS by silybin was partially attenuated by inhibitors of NF-κB or protein kinase C (PKC), suggesting an involvement of NF-κB and PKC signaling pathways. Similarly, a significant decrease in NF-κB was observed, when PBMCs from pre-eclamptic patients were cultured with silibinin at 5 μM and 50 μM concentrations and stimulated with LPS [215]. Moreover, silibinin (50–200 μM) inhibited the nuclear translocation of nuclear factor NF-κB through inhibition of the phosphorylation of IκBα and suppressed NF-κB transcriptional activity in stimulated HMC-1 cells [216]. SM is also effective in inhibiting T cell activation and proliferation, by acting on pathways of NF-κB activation/translocation. In particular, in CD4+ splenocytes from C57/Bl6 mice, SM (50 μM) significantly inhibited CD4+ cells proliferation, inhibited IL-2 and IFN-gamma production and blocked nuclear translocation of transcription factor NF-κB. Moreover, SM inhibited p65/NF-κB phosphorylation in CD4+ T cell [217]. The significant upregulation of oxidative stress biomarkers including MDA, TNF-like, IFN-γ and IL-1β genes was observed as well as NF-κB, COX-2 and iNOS proteins expression occurred upon heat stress in chicken hepatocytes. Furthermore, AO enzyme (SOD, CAT, GR) activities decreased. SM (259 μM) was able to normalize the expression of all of these biomarkers in heat-induced chicken hepatocytes [218]. However, there is a need for more studies with pure silybin at physiologically-relevant concentrations (0.2–2 μM) in order to confirm its modulating effect on the NF-κB pathway.

##### 5.2.1.2. *In Vivo* Studies

It has been shown that silibinin (50 mg/kg/BW) had a protective effect against d-galactose-induced senescence due to promotion of cellular oxidoreductase activities and inhibition of NF-κB activation and ROS production [219]. Histopathological and immunohistochemical studies in the kidney of rats also shows that silibinin (75 mg/kg/BW) markedly reduced the toxicity of As and preserved the normal histological architecture of the renal tissue, inhibited the caspase-3 mediated tubular cell apoptosis and decreased the NADPH oxidase, iNOS and NF-κB overexpression by As [72]. In a model of experimental ischemic stroke in male rats, silibinin (100 mg/kg) significantly downregulated NF-κB in ischemic brain tissue after stroke [189]. In an experimental nonalcoholic steatohepatitis model, mice fed a methionine-choline deficient diet consistently had increased NF-κB p65 and p50 binding activity, while silibinin administration (20 mg/kg/BW/i.p.) significantly decreased the activity of both subunits [220]. It was shown that CCl4 treatment of mice enhanced the NF-κB expression in the liver and induced hepatic fibrosis, while SM (200 mg/kg/BW) reduced the CCl_4_-induced hepatic NF-κB and improved CCl_4_-induced liver fibrosis [88]. Protective effect of silibinin (150 mg/kg/BW/intragastric) in the rat model of cerebral ischemia was associated with decreased expression of the Bax, NF-κB protein and Bax, NF-κB mRNA in the brain [221]. SM is also shown to have NF-κB inhibiting effects. For example, mRNA and protein expressions of NF-κB were significantly up-regulated in d-galactosamine induced hepatotoxic rats and treatment with SM (25 mg/kg/BW) significantly down-regulated the expressions of these genes [222]. Furthermore, it has been shown that the serum levels of TGF-β1, NF-κB, and IL-6 were significantly elevated in the samples from cirrhosis rats while SM (50 g/kg) significantly prevented the aforementioned changes [223] and SM (250 mg/kg/BW) suppressed NF-κB signaling cascade in alcoholic liver fibrosis in guinea pigs [224,225]. Overall, SM/silibinin targets multiple signaling pathways, including NF-κB, towards inhibiting the secretion of pro-inflammatory cytokines. Together these findings suggest that the inhibitory effect of SM/silibinin on NF-κB signaling in a variety of cells, as well as in *in vivo* studies, could be an important mechanism of its anti-inflammation efficacy, and that targeting this signaling pathway by silibinin may have potential applications in the clinic and veterinary practice.

## 6. Effect of SM on Vitagene Expression

The term “vitagene” was introduced in 1998 by Rattan [226] who wrote “Our survival and the physical quality of life depends upon an efficient functioning of various maintenance and repair processes. This complex network of the so-called longevity assurance processes is composed of several genes, which may be called *vitagenes*”*.* Later, the vitagene concept has been further developed by Calabrese and colleagues [53,227,228,229,230,231,232,233,234,235,236,237] and major pro-survival mechanisms controlled by homodynamic vitagene network are shown in Table 1. In accordance with Calabrese *et al.* [227,228,229,230,231,232,233,234,235,236,237,238] the term vitagenes refers to a group of genes that are strictly involved in preserving cellular homeostasis during stress conditions and the vitagene family includes heat shock proteins (Hsps), heme oxygenase-1 (Hsp32, HO-1), Hsp60 and Hsp70, the thioredoxins (Trx)/thioredoxins reductase (TR) system and sirtuins. The list of potential candidates to the vitagene family can be extended. In particular, it seems likely that SOD, a major inducible enzyme of the first level of antioxidant defence, can meet selecting criteria to be included in the vitagene family. The products of the aforementioned genes actively operate in detecting and controlling diverse forms of stress and cell injuries. The cooperative mechanisms of the vitagene network are reviewed in recently published comprehensive reviews [234,237,238] with a major conclusion indicating an essential regulatory role of the vitagene network in cell and whole organism adaptation to various stresses.

Emerging findings suggest a large number of potential mechanisms of action of SM (polyphenols) in preventing disease, which may be beyond their direct conventional antioxidant activities. It seems likely that SM, similar to other flavonoids, can affect the vitagene network. The recent relevant findings are reviewed below. First of all, SM/silybin affects HO-1 activity in different model systems. For example, As-intoxicated rats showed a significant up-regulation of myocardial NADPH (NOX) oxidase sub-units such as NOX2 and NOX4 as well as Keap1 and down-regulation of Nrf2 and vitagene HO-1 protein expressions. Pre-administration of silibinin (75 mg/kg/BW) recovered all these altered parameters to near normalcy in As-induced cardiotoxic rat [71]. Similarly, in a model of liver injury caused by alcohol plus pyrazole, SM administration (50 mg/kg/BW) had a protective effect with a trend in restoring the decreased activity of HO-1 and Nrf2 [183]. SM (250 mg/kg/BW) possesses substantial protective effect against B(a)P-induced damages by increasing (restoring) HO-1 (vitagene) activity [115]. Similarly, *in vitro* SM (500 μM) reduced tBH-induced hepatocyte toxicity by activating HO-1 gene expression [239]. Indeed, the enzyme HO-1 is an important regulatory molecule present in most mammalian cells. In fact, the main function of HO-1 is to break down the pro-oxidant molecule heme into three products; carbon monoxide (CO), biliverdin and free iron and actively participate in the antioxidant defence in the human/animal body [240]. Indeed, HO-1 is a stress-inducible protein and can be induced by various oxidative and inflammatory signals. Therefore, HO-1 expression is regarded as an important adaptive cellular mechanism of AO defence in stress conditions [241,242].

**Table 1:** Major components of the vitagene network (adapted from [226] and [228]).

Molecular Level	Cellular Level
AO defense	Cell proliferation
DNA-repair systems	Cell differentiation
Transfer of genetic information	Stability of cell membrane
Stress protein synthesis	Stability of intracellular milieu
Proteosomal function	Macromolecular turnover
**Tissue and Organ Level**	**Physiological and Redox Control Level**
Neutralization and removing toxic chemicals	Neuronal response and synaptic plasticity
Tissue regeneration and wound healing	Stress response
Tumor suppression	Hormonal response
Cell death and cell replacement	Immune response
	Thermoregulation
	HO-1/CO; BVR/BR; UCP; Hsp70; Hsp27; TRXr/TRX

From the data presented above it is clear that SM/silibinin can upregulate HO-1 and improve antioxidant defences. Secondly, SM/silibinin can affect other Hsps including Hsp70. Indeed, in an *in vitro* system based on CHO-K1 cells treated with As, SM (5 μM) significantly decreased Hsp70 expression previously elevated by As [243]. In another *in vitro* system based on heat-induced chicken hepatocytes, SM (259 μM) affected Hsp70 expression significantly, preventing its alleviation by heat stress [218]. A similar protective effect of SM (100 mg/kg/BW) on Hsp70 was seen in rats given SM for 7 days prior to mesenteric ischemia-reperfusion (I-R) compared to I-R group [244]. It is interesting to note that silybin was identified as a novel Hsp90 inhibitor [245]. Therefore, silibinin can decrease Hsp70 expression in stressed cells indicating improved AO defences and decrease stress by other means (e.g., Nrf2-related increased AO synthesis).

Thirdly, there are several recent publications showing a regulatory role of SM/silibinin on the sirtuin functions. In fact, a protective effect of silibinin against chemotherapeutic reagent mitomycin C-induced cell death in A375-S2 cells was studied. It was shown that mitomycin C caused cell apoptosis and over-expression of p53, elevated translocation of p53 into the nucleus, and decreased SIRT1 expression; and these effects were alleviated by silibinin (100–150 μM) to a large extent [246]. Silibinin protected cardiac myocytes against isoproterenol-induced injury through resuming mitochondrial function and regulating the expression of SIRT1 and Bcl-2 family members [247]. Systemic administration of silibinin (50 mg/kg/BW) reversed streptozotocin-induced downregulation of SIRT1 expression in mice [248]. It is important to note that SIRT1 was activated by SM (500 μM) in UV-irradiated human malignant melanoma, A375-S2 cells [249]. However, silibinin could have completely the opposite action in regulating SIRT1 expression in normal cardiac myocytes and breast cancer MCF-7 cells, where it downragulates SIRT1 and promotes apoptosis [250]. Similarly silybin treatment downregulated SIRT1 in human lung adenocarcinoma cells [251].

Recent findings indicate that sirtuins-mediated signaling pathways are involved in metabolic reprogramming and upregulation of antioxidant defense systems [252]. Sirtuins are a class of proteins possessing deacetylase activity and playing critical roles in cell survival in response to oxidative stress. Seven human sirtuins, SIRT1–SIRT7, have been identified and their localization differ considerably: SIRT1 and SIRT2- nucleus and cytoplasm; SIRT3, SIRT4 and SIRT5- mitochondria; SIRT3- nucleus and cytoplasm and SIRT6 and SIRT7- nucleus [253]. Sirtuins control DNA repair and recombination, chromosomal stability and gene transcription, as well as regulate antioxidant defences. On the one hand, sirtuins decrease ROS levels, inhibit apoptosis and decrease inflammation. On the other, oxidative stress has been shown to decrease SIRT1 expression in the rat hippocampus and cortex, while SIRT1 overexpression prevents oxidative stress-induced apoptosis and increases resistance to oxidative stress (for review see [254]). Sirtuins are responsible for deacetylation of important transcription factors such as p53, FoxO, NF-κB, or PAPARGCα, which control the transcription of pro- and antioxidant enzymes [255]. Therefore, SM-related activation or prevention of inhibition of sirtuins in stress conditions could be considered as an important adaptive mechanism responsible for maintaining redox-regulated homeostasis in the cell and in the whole body. Finally, there is only a single publication devoted to the protective effect of SM on another vitagene called thioredoxins system. Silybin-phospholipid complex containing vitamin E (Realsil^®^, Indena, IBI-Lorenzini spa, Italy) was administered daily by gavage (15 mg vitamin E and 47 mg silybin complexed with phospholipids) to rats fed a choline-deprived or a high fat diet for 30 days and 60 days, respectively. Realsil was shown to be able to maintain Trx concentration in the rat liver fed on a high fat diet at day 60, while in the control group it dramatically decreased [59]. It should be mentioned that Trx, a small, ubiquitous thiol protein, is one of the most important regulators of redox balance and redox-sensitive cell functions. In fact, the thioredoxin system consisting of Trx, thioredoxin reductase (TrxR), and NADPH, is a key element determining cell redox status and regulating protein dithiol/disulfide balance, signal transduction and gene expression [256]. Indeed, the Trx system together with the glutathione-glutaredoxin (Grx) system (NADPH, glutathione reductase, GSH, and Grx) controls the cellular redox environment and determines a variety of cellular functions including DNA metabolism and repair, transcription, intracellular signaling, cell-cell communication, cell growth, apoptosis inhibition and ultimately cell survival in various stress conditions [256,257]. It is well accepted that the ability of SM to maintain Trx system in stress conditions is an important element of improving the adaptive ability of animals/humans via the vitagene network. Therefore, the vitagene network represents the major cellular pathway involved in the so called “programmed cell life” as an opposite to apoptosis, providing an effective protection against oxidative stress and toxic products of ROS metabolism [230,231,232]. In particular, nutritional antioxidants can be neuroprotective through the activation of hormetic pathways under the control of the Vitagene protein network [234]. The aforementioned results clearly indicate that, similar to other flavonoids SM, or its active ingredient silibinin, affect vitagene network decreasing detrimental consequences of stresses. Further research is needed to fully understand the molecular mechanisms involved in the vitagene network activation by SM/silybin.

## 7. Protective Effect of SM in the Gut

It seems likely that the gut is the major place of antioxidant action of polyphenols [258,259]. Indeed, reduction of oxidative damage, modulation of colonic flora and variation in gene expression are involved in the modulation of intestinal function by polyphenols. For example, to study the molecular effects of wine polyphenols at the gene level, the microarray technology was used: rats were treated with 50 mg/kg/BW wine polyphenols for 14 days. Global expression analysis of 5707 genes revealed an extensive down-regulation of genes involved in a wide range of physiological functions, such as metabolism, transport, signal transduction and intercellular signaling [260]. It was shown that two major regulatory pathways were down-regulated in the colon mucosa of polyphenols treated rats: inflammatory response and steroid metabolism. Since flavonoids are consumed in concentrations usually much higher than other antioxidant compounds, their protective effect during digestion is of great importance. For example, it has been shown that flavonoids not only prevented an accumulation of peroxidized lipids but can also switch prooxidant properties of heme-proteins to antioxidant ones [261]. Dietary polyphenols can also modulate *in vivo* oxidative damage in the gastrointestinal tract of rodents [262] supporting the hypothesis that dietary polyphenols might have both a protective and a therapeutic potential in oxidative damage-related pathologies. Indeed, the antioxidant-prooxidant balance (redox status) in various parts of the intestine would ultimately depend on the level of antioxidants and pro-oxidants provided by the diet and released by cells themselves, as well as on the level of absorption of both antioxidants and pro-oxidants [258,259]. In a model system mimicking stomach conditions it was shown that both lipid peroxidation and co-oxidation of vitamin E and beta-carotene were inhibited at pH 3.0 by red wine polyphenols [263]. In the gut, an interaction between different antioxidants could bring about their maximum synergistic effects. In particular, it was found that the mixture of the green tea polyphenol, vitamin E and vitamin C could act synergistically to protect lipid peroxidation [264]. It has been found that consumption of partially oxidized food could increase lipid peroxidation in the stomach and the absorption of cytotoxic lipid peroxidation products into the body. The addition of red wine polyphenols to the meal may alter these outcomes [265], therefore red wine polyphenols exert a beneficial effect by the novel function, absorption inhibition of the MDA [266]. These findings explain the potentially harmful effects of oxidized fats found in foods/feeds [259] and the important benefit of dietary polyphenols in the diet. Indeed, locating the biological site of action of polyphenols in the gastrointestinal tract may lead to a revision in our understanding of how flavonoids, including silybin, work *in vivo* and may help to elucidate the mechanisms of the health-promoting action of silybin and other polyphenols [267].

Redox signaling in gut inflammation is complex and poorly understood. However, it is generally accepted that homeostatic control of the intestinal epithelial redox environment is central for nutrient digestion and absorption, stem cell proliferation, apical enterocyte apoptosis, and immune response [268]. Indeed, polyphenols may play a role in intestinal mucosa integrity, inflammation and permeability [258,269]. For example, wine phenolics were able to prevent or delay the progression of intestinal diseases characterized by oxidative stress and inflammation, acting as both free radical scavengers and modulators of specific inflammation-related genes involved in cellular redox signaling [270]. They exert their effects by modulating cell signaling pathways, mainly activated in response to oxidative and inflammatory stimuli, and Nrf2 and NF-κB are the principal downstream effectors [271]. For example, pigs administered a grape seed and grape marc extract had a lower transactivation of NF-κB and Nrf2 and a lower expression of various target genes of these transcription factors in the duodenal mucosa (but not in the liver and plasma) than control pigs [272]. Furthermore, the ratio of villus height: crypt depth and the gain:feed ratio was higher in the animals fed a gape extract than in control pigs. In mice, the dietary grape seed extract decreased proliferation and enhanced differentiation of epithelial cell and the changes in gut epithelium were associated with the suppression of NF-κB signaling [273]. Polyphenolic compounds could also affect gut integrity. For example, grape seed extract (0.1%) supplied to rats with drinking water for 21 days significantly increased the expression of gut junction protein occludin in the proximal colon and reduced fecal levels of the neutrophil protein calprotectin, compared with control [274]. Recently, it has been reported that a naturally occurring flavonoid procyanidin B2 could modulate cellular redox status and the antioxidant enzyme defence system in colonic cells protecting against oxidative stress and xenobiotics [275,276].

It is possible to suggest that there is a biological reason for some antioxidants not to be absorbed completely and so provide antioxidant protection in lower parts of the intestine [258,259]. Comparatively low bioavailability and antioxidant potential of various flavonoids could be beneficial for humans/animals providing antioxidant protection in various parts of the digestive tract, including the large intestine where levels of other antioxidants would be quite low. The same is true for SM. For example, silibinin concentration in the gut could reach 800 μM [277], a concentration exceeding the requirement for its direct antioxidant activity. Indeed, it is still unclear whether SM has any direct antioxidant effects *in vivo*, although it might be capable of exerting such effects within the gastrointestinal tract, where SM may come into direct contact with cells without having undergone absorption and metabolism [258]. It has been suggested that gastroprotective effects of SM might be related to the protection of gastric mucosal NO and non-protein sulfhydryl groups and the modulation of capsaicin-sensitive gastric sensory afferents [278]. The beneficial effects of SM, on a murine model of colitis was explained [279]. SM effectively reduced colonic damage, lipid peroxidation, neutrophil infiltration and the content of inflammatory cytokines alongside increasing total antioxidant capacity of colonic tissue. In trinitrobenzene sulfonic acid-induced colitis model, silibinin treatment significantly reduced several components of inflammatory colitis such as NF-κB activity, levels of IL-1β, TNFα, TBARS, protein carbonyl, myeloperoxidase activity, and an improvement in antioxidant capability of the colon tissue [280]. Furthermore, silibinin (50 mg/kg/BW) modulates gut microbial enzymes, colonic oxidative stress and Wnt/β-catenin signaling resulting in antiproliferative effect against 1,2-di-methylhydrazine-induced colon carcinogenesis in experimental rats [64].

It is also well known that polyphenols are providing antioxidant protection in the lower intestine and can alter colonic flora [281]. Polyphenols, including silybin, are extensively metabolized by gut bacteria into a complex series of end-products that affect the functional ecology of symbiotic partners that can alter the host physiology [281]. It has been suggested that the consumption of a diet rich in plant foods with high dietary polyphenol content may enhance the gastrointestinal health of the host through microbiota modulation. Indeed, grape antioxidant dietary fiber intake stimulates proliferation of *Lactobacillus* and slightly affects the composition of *Bifidobacterium* species in the cecum of rats [282]. It appears that dietary polyphenols may have the ability to modify the gut microbial balance, but this effect is indirect, *i.e.*, it is mediated by biotransformation products, rather than the original plant compounds [283]. It should be mentioned that polyphenol–microbiota interactions are complex and subject to large inter-individual variability, leading to different polyphenol-metabolizing phenotypes or “metabotypes” [284].

Unfortunately, there is no data available on the effect of SM on gut microbiota, but we can suggest the same changes would occur as described above for other flavonoids. Therefore, it seems likely that reduction of oxidative damage, decreased inflammation, modulation of colonic flora and variation in gene expression are involved in the modulation of intestinal function by polyphenols, including SM.

## 8. Conclusions

Recent achievements in biochemistry and molecular biology, together with epidemiological data have changed our thinking about food. It has become increasingly clear that our diet plays a pivotal role in maintenance of our health and a misbalanced diet can cause serious health-related problems. It seems likely that antioxidants are among the major regulators of many physiological processes and, therefore, a redox balance between antioxidants and prooxidants in the diet, gastro-intestinal tract, plasma and tissues is an important determinant of the state of our health. Plants consumed by humans amd animals contain thousands of phenolic compounds. Among them, the effects of dietary polyphenols including SM are of great current interest. Indeed, various phytochemicals, including flavonoids, are an essential part of our diet, which are responsible for turning on and maintaining an optimal status of our antioxidant defences. Since flavonoids are not well absorbed in the gut, their active concentration in the plasma and target tissues are comparatively low, but probably sufficient for Nrf2 activation and NF-κB suppression as well as vitagene activation. Indeed, it seems very likely that activation of the Keap1/Nrf2/ARE pathway and inhibition of NF-κB pathway, rather than direct free radical scavenging activity, may be the main mechanisms of the health benefits of phytochemicals [233], including SM. Therefore, consumption of phytochemicals, including SM, could have a pre-conditioning effect on the antioxidant system of the body. This could explain the beneficial health-promoting effects of a diet rich in fruits and vegetables as important sources of the aforementioned chemicals (polyphenols and other phytochemicals) maintaining the body’s ability to be highly adaptive to various stresses. SM and its main component silibinin are part of the dietary phytochemical mixture responsible for regulation of the antioxidant defences in the gut and in the whole body. It could well be that some dietary constituents which are not well absorbed could have health-promoting properties by maintaining redox balance in the large intestine, where concentration of other antioxidants (vitamin E, carotenoids, ascorbate) could be low, but prooxidants (iron, oxidized PUFAs, *etc.*) and substrates of oxidation are still present [258,259]. This protective effect in the large intestine could be responsible, for example, for bowl cancer prevention. Therefore, there could be a biological reason for some nutrients not being absorbed, but still being involved in antioxidant protection in the lower gut. Taking into account high concentrations of phytochemicals in the gut, it could well be that they play an essential part in maintaining an optimal antioxidant-prooxidant balance in the digestive tract responsible for additional health effects of phytochemicals including SM. In animal nutrition and disease prevention strategy SM alone, or in combination with other hepatho-active compomds (carnitine, betaine, vitamin B12, *etc.*), could have similar hepatoprotective effects as described in humans with similar mechanisms of protective action.

In conclusion, there are many possible mechanisms by which SM can improve the antioxidant defence mechanisms in the body. They include direct and indirect SM actions. First of all, a direct scavenging free radicals and chelating free Fe and Cu are mainly effective in the gut. Secondly, preventing free radical formation by inhibiting specific ROS-producing enzymes, or improving the integrity of electron-transport chain of mitochondria in stress conditions as a result of SM consumption, is of great importance. Thirdly, maintaining an optimal redox balance in the cell by activating a range of antioxidant enzymes and non-enzymatic antioxidants, mainly via Nrf2 activation, is probably the main driving force of AO action of SM. Fourthly, decreasing inflammatory responses in the gut and other tissues by inhibiting NF-kB pathways is an emerging mechanism of SM protective effect in liver toxicity and diseases. Fifthly, activating vitagenes, responsible for synthesis of protective molecules, including HSP, Trx, sirtuins, *etc.*, and providing additional protection in stress conditions deserves more attention in future research. Finally, affects on the microenvironment of the gut, including SM-bacteria interactions, await future investigation.
